# Making sense of IL‐6 signalling cues in pathophysiology

**DOI:** 10.1002/1873-3468.14201

**Published:** 2021-10-15

**Authors:** David Millrine, Robert H. Jenkins, Stuart T. O. Hughes, Simon A. Jones

**Affiliations:** ^1^ Division of Infection & Immunity School of Medicine Cardiff University UK; ^2^ Systems Immunity University Research Institute Cardiff University UK; ^3^ Present address: Medical Research Council Protein Phosphorylation and Ubiquitylation Unit Sir James Black Centre School of Life Sciences University of Dundee 3rd Floor Dundee UK

**Keywords:** arthritis, cytokine receptors, cytokines, epigenetics, inflammation, interleukin, Jak‐STAT signalling, microRNA, pathophysiology, STAT transcription factors

## Abstract

Unravelling the molecular mechanisms that account for functional pleiotropy is a major challenge for researchers in cytokine biology. Cytokine–receptor cross‐reactivity and shared signalling pathways are considered primary drivers of cytokine pleiotropy. However, reports epitomized by studies of Jak‐STAT cytokine signalling identify interesting biochemical and epigenetic determinants of transcription factor regulation that affect the delivery of signal‐dependent cytokine responses. Here, a regulatory interplay between STAT transcription factors and their convergence to specific genomic enhancers support the fine‐tuning of cytokine responses controlling host immunity, functional identity, and tissue homeostasis and repair. In this review, we provide an overview of the signalling networks that shape the way cells sense and interpret cytokine cues. With an emphasis on the biology of interleukin‐6, we highlight the importance of these mechanisms to both physiological processes and pathophysiological outcomes.

## Graphical abstract

The interleukin‐6 signalling cassette controls cytokine activities essential for homeostasis, immune defence and disease progression. In this review, we discuss the regulatory processes determining IL‐6 bioactivity and bioavailability. We consider the intracellular events that orchestrate and fine‐tune the delivery of Jak‐STAT signals and explore the transcriptional mechanisms that dictate the interpretation of IL‐6 signals in physiology and pathophysiology.

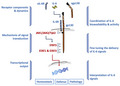

## Abbreviations

**DUSP**, dual specificity protein phosphatases

**ELS**, ectopic lymphoid‐like structures

**GAS**, gamma activated sequence

**HDL**, high‐density lipoprotein

**IL**, interleukin

**ISRE**, interferon stimulated responsive element

**Jak**, Janus activated kinase

**miR**, microRNA

**PIAS**, protein inhibitor of activated STAT factors

**PTP**, protein tyrosine phosphatase

**SNP**, single nucleotide polymorphis

**SOCS**, suppressor of cytokine signalling

**STAT**, signal transducer and activator of transcription

Interleukin (IL)‐6 controls multiple phenotypic traits that impact both immune effector functions and the regulation of homeostatic processes. Although traditionally viewed as a pro‐inflammatory cytokine involved in disease progression, the biology of IL‐6 is complex contributing to metabolism, development, tissue turnover and psychological well‐being [1, 2, 3, 4, 5, 6, 7, 8]. This range of cytokine activities illustrates the context‐dependent nature of IL‐6 signalling. How IL‐6 acting through a single receptor system elicits these diverse biological outcomes remains unclear. So, what is the evidence for IL‐6 involvement in these processes?

The functional pleiotropy of IL‐6 is typified by observations made in patients lacking a functional IL‐6 receptor cassette or downstream signalling intermediates, and children with anti‐IL‐6 autoantibodies [9, 10, 11, 12, 13, 14, 15]. These individuals show altered humoral and T‐cell responses, eosinophilia, impaired acute phase response, susceptibility to fungal infection, epidermal afflictions including eczema and skin lesions suggestive of connective tissue defects [9, 10, 11, 12, 13, 14]. Similar phenotypic traits are also seen in patients with mutations in STAT1 and STAT3, the principle downstream transcription factors of IL‐6 signalling [9, 16, 17]. Other examples have arisen from documentation of the clinical efficacy of biological drugs and small molecule inhibitors that block the IL‐6 pathway [1, 4, 18]. These include monoclonal antibodies that target IL‐6 (e.g. clazakizumab, olokizumab, sirukumab, siltuximab, ziltivekimab) or prevent IL‐6 binding to the cognate IL‐6 receptor (e.g. tocilizumab, sarilumab), and Janus kinase inhibitors such as tofacitinib [1, 2, 3, 4, 5, 6, 7]. For instance, the failure of anti‐IL‐6 therapy in inflammatory bowel diseases is often cited as an example of IL‐6 functional complexity. Here, its immunomodulatory activities are weighted against its contribution to the maintenance of barrier integrity in epithelial tissues [1, 4]. IL‐6 is also essential for the regulation of lipid, glucose and iron metabolism, and is integral to the control of mitochondrial bioactivity [1, 2, 4, 18]. These activities impact processes affecting appetite, fatigue and energy expenditure. For example, IL‐6 reduces appetite, delays gastric emptying and regulates postprandial glycaemia and adiposity [18, 19, 20]. High cholesterol, increased serum levels of high‐density lipoprotein (HDL), and weight gain, are all reported consequences of tocilizumab intervention [1, 2, 4, 21]. However, IL‐6 antagonism changes the lipid composition to lower cholesterol‐associated proatherogenic factors, such as HDL‐associated serum amyloid‐A and secretory phospholipase‐A2 [21, 22]. Similar studies also identify prominent roles for IL‐6 in pain perception, psychological well‐being, the regulation of haematopoiesis, and processes affecting tissue turnover, regeneration and repair [8, 18, 23, 24, 25, 26]. How IL‐6 signalling intermediates converge to regulate these homeostatic processes while at the same time supporting host defence and the response to tissue damage is unclear. These uncertainties have complicated the use of IL‐6 targeting therapeutics in infectious diseases including bacterial peritonitis, sepsis and the recent COVID‐19 public health emergency [27].

Significantly, studies in drosophila and mice re‐affirm the dynamic and pleiotropic properties of IL‐6 in physiology and pathophysiology [28]. Here, the application of cytokine and cytokine receptor‐deficient mice, genetic knock‐in strains and pharmaceutical agents (e.g. antibodies, soluble receptors and engineered fusion proteins) have helped to frame the involvement of IL‐6 in health and disease [1, 2, 4, 29, 30, 31]. While these studies have pioneered the development of biological drugs routinely used in clinical practice to inhibit IL‐6, they have also identified the mechanistic involvement of IL‐6 signalling in exercise, pain, anhedonia, appetite, metabolism and tissue homeostasis [18]. Thus, the biological activities of IL‐6 are highly conserved across species and experimental model systems provide exciting opportunities to understanding the signalling basis of IL‐6.

## The IL‐6 signalling cassette

Almost all stromal cells (e.g. endothelial cells, fibroblasts) and certain subsets of immune cells (e.g. macrophages and other monocytic cells) produce IL‐6 in response to Toll‐like receptor agonists, cytokines (e.g. TNFα, IL‐1β, IL‐17, GM‐CSF), lipid mediators (e.g. prostaglandins), adipokines, and as a consequence of cellular stress. Significantly, IL‐6 gene expression is subject to both homeostatic control and rapid induction following inflammatory challenge as a response to infection, trauma, autoimmunity or cancer. These involvements are equally reflected by the definition of IL‐6 as an adipokine, myokine, neurotrophic factor, lymphokine and monokine. How IL‐6 coordinates each of the activities is subjected to much research, and the complex nature of IL‐6 biology is highlighted by the various signalling mechanisms adopted by cells to sense and interpret changes in IL‐6 bioactivity (as discussed below).

**Fig. 1:**
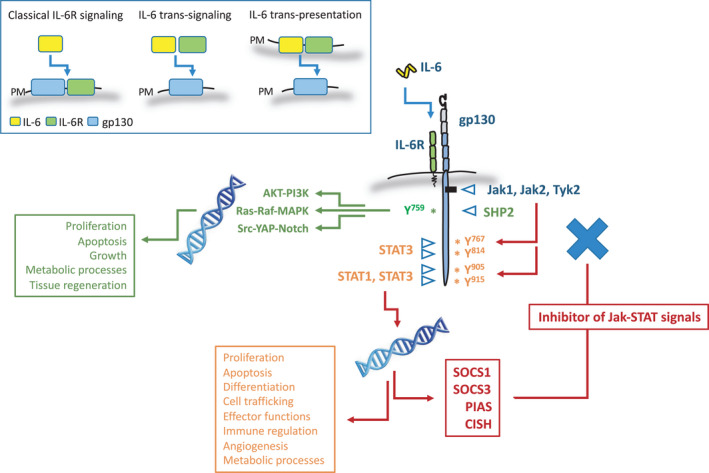
Modes of IL‐6 signalling and determinants of IL‐6 responsiveness. The boxed cartoon representations show the three modes of IL‐6 receptor signalling. Subunits of the cytokine receptor cassette are colour coded as indicated. PM denotes plasma membrane, and shows the subunits bound to the cell surface in each signalling mode. The wider panel shows how the signalling intermediates associated with IL‐6 receptor signalling engage with gp130 (tyrosine residues based on the human gp130 sequence are designated) and contribute to the cellular activities of IL‐6. Note, the expression of STAT‐inducible negative feedback inhibitors, which act to limit the protracted activation of the receptor complex.

IL‐6 signals through at least three distinct mechanisms termed classical IL‐6R signalling, IL‐6 trans‐signaling and IL‐6 trans‐presentation (Fig. 1) [4]. The classical signalling cascade begins when IL‐6 binds the cognate IL‐6 receptor (IL‐6R; also known as CD126). The IL‐6R represents a type‐1 cytokine receptor and as the α‐subunit of the IL‐6 receptor complex it is responsible for sensing changes in IL‐6 bioactivity [32, 33, 34]. IL‐6R is non‐signalling by nature [32, 33, 34]. Once engaged by IL‐6, the IL‐6R complexes with gp130, triggering receptor oligomerization and formation of a functioning receptor complex composed of two IL‐6‐IL‐6R‐gp130 trimers (Fig. 1) [34, 35, 36, 37, 38]. Two gp130 molecules engage with one another in an interlocking structure wherein each gp130 molecule contacts two molecules of IL‐6 *via* separate interfaces [39, 40, 41]. Receptor cross‐linking renders gp130 signalling competent through a conformational change that allows Janus kinases (namely Jak1, Jak2 and Tyk2) to catalyse the phosphorylation of tyrosine residues within the cytoplasmic domains of gp130 [4, 31, 32, 33]. These sites act as docking regions for the STAT transcription factors STAT1, STAT3 and to a lesser extent STAT5 [32, 33, 42, 43, 44]. This activates the phosphorylation‐induced dimerization of previously latent STAT transcription factors, which translocate to the nucleus to exert their regulatory functions on gene expression [45]. Beyond the regulation of Jak‐STAT signalling, gp130 also transmits signals through the SHP2 cascade [32, 33, 46].

This receptor system is elegantly poised to contain or limit the level of IL‐6 receptor signalling. Here, various mechanisms have evolved to restrain gp130‐mediated outcomes. For example, regulatory processes that affect receptor internalization and deactivation and signalling intermediates that inhibit or modify the signalling output of IL‐6. Many will be discussed in due course, but include protein tyrosine phosphatases (PTPs), microRNA (miR) that target mRNAs encoding components of the IL‐6 receptor cassette, and STAT‐inducible factors that act as negative feedback inhibitors of gp130 signalling (e.g. PIAS, SOCS1, and SOCS3) [45, 46, 47, 48]. Thus, a wide range of intracellular processes manage the way IL‐6 signals are sensed and interpreted by cells. However, before delving into these processes, we must first consider the extracellular events governing IL‐6 bioavailability and bioactivity.

## IL‐6 bioactivity and bioavailability

Although gp130 was initially identified as the signalling subunit of the IL‐6 receptor complex, this glycoprotein displays a broad pattern of cellular expression and functions as the β‐cytokine receptor for IL‐11, IL‐27, oncostatin‐M, ciliary neurotrophic factor, leukaemia inhibitory factor, cardiotrophin‐1 and cardiotrophin‐like cytokine [3, 4, 29]. Mice lacking *gp130* (also referred to as *Il6st*) are embryonically lethal with genetic mutation studies identifying essential roles for gp130 in development, haematopoiesis, tissue homeostasis, cell survival and growth, and immune regulation [3, 29]. In contrast, IL‐6R expression is more restricted and confined to leukocytes, hepatocytes, megakaryocytes and some mesenchymal populations [3, 29]. As a consequence, it is often challenging to understand how IL‐6 delivers its full repertoire of biological activities. This significantly changed with the discovery of a soluble IL‐6R (sIL‐6R) in human urine and plasma [49, 50]. IL‐6 binds sIL‐6R to form an agonistic complex capable of triggering gp130 signalling (termed IL‐6 trans‐signalling) [29, 49, 50, 51]. Sharing sequence identity with IL‐12p40 and EBI3, the IL‐6‐sIL‐6R complex resembles a heterodimeric cytokine (akin to IL‐12, IL‐23, IL‐27) that is capable of broadening the types of cells that are responsive to IL‐6 [52, 53]. The current view is that IL‐6 trans‐signalling primarily shapes immune responses essential for host defence and processes affecting tissue injury or pathology, whereas classical IL‐6R signalling coordinates the more physiological aspects of IL‐6 bioactivity [3, 4, 29, 30]. This paradigm is supported by extensive experiments in murine models of inflammation using soluble gp130 (sgp130), a pathway inhibitor with specificity for IL‐6 trans‐signalling and IL‐11 trans‐signalling [1, 2, 3, 29, 54, 55]. For example, transgenic mice over‐expressing sgp130 are protected from acute inflammatory challenge [56].

In human serum, sgp130 is present at saturating levels (100–400 ng·mL^−1^) where it constitutes an IL‐6 buffering system that prevents penetrance of minor fluctuations in IL‐6 concentration being interpreted as a danger signal [1, 2]. These potential systemic consequences of IL‐6 trans‐signalling may explain the need for a third mode of signal transduction [2, 57, 58]. IL‐6 trans‐presentation (also termed cluster signalling) describes the initiation of signalling resulting from cell‐cell interactions between IL‐6R‐expressing antigen presenting cells and gp130 on T cells [2, 58]. IL‐6‐IL‐6R complexes are generated intracellularly by dendritic cells and presented on the cell surface to gp130‐expressing T cells. This juxtacrine‐type interaction drives STAT3 signalling and Th17 polarization. This process is critical for Th17 mediated inflammation of the central nervous system in murine experimental autoimmune encephalomyelitis [58]. Although the pathological implications of IL‐6 trans‐presentation are yet to established in humans, it is expected to be refractory to anti‐IL‐6R targeting biologics and may, therefore, represent a novel opportunity for blockade of IL‐6 activities [2, 30].

The coordination of these modes of IL‐6 receptor signalling is influenced by changes in the bioavailability of IL‐6, sIL‐6R and sgp130. IL‐6 expression is tightly regulated at the transcriptional level to ensure physiological fluctuations in IL‐6 during homeostasis and rapid induction during infection, trauma or injury [1, 18]. In humans, circulating IL‐6 levels remain low (1–5 pg·mL^−1^), but these concentrations oscillate during the course of the day and are equally subject to seasonal variations between summer and winter months [59, 60, 61, 62, 63, 64]. Following immune activation, serum IL‐6 levels are rapidly increased (frequently reaching ng·mL^−1^ or μg·mL^−1^ quantities) in response to cytokines, Toll‐like receptor agonists, prostaglandins and other stress signals [1, 4, 65]. Here, certain miR (e.g. Let‐7a), ribonucleases (e.g. regnase‐1) and other RNA‐binding factors (e.g. Lin28B, Arid5a) function to regulate IL‐6 gene expression [1]. At the genetic level, various single nucleotide polymorphisms (SNPs) affect the bioactivity or bioavailability of IL‐6 [66, 67, 68]. For example, rs1800795, resulting in a G‐to‐C mutation in the transcriptional promoter of the IL‐6 gene causes alterations in *IL6* expression [69, 70]. Carriers of this mutation display increased risk for coronary heart disease, idiopathic juvenile arthritis and other forms of inflammatory disease [70, 71, 72]. Similar phenotypic traits are also seen in individuals with either *IL6R* or *IL6ST* polymorphisms [73, 74]. Individuals carrying the *IL6R* polymorphism rs2228145 show enhanced sIL‐6R and IL‐6 levels, reduced C‐reactive protein and a lower risk of cardiovascular disease [75, 76]. Conversely, these individuals show protection against SARS‐CoV‐2 disease and present with a reduced likelihood of hospitalization as a consequence of severe infection [27, 68, 77].

A challenge for IL‐6 researchers is to understand the dynamics and kinetics of IL‐6 bioactivity. For example, what is the physiological role for classical IL‐6R signalling versus IL‐6 trans‐signalling and IL‐6 trans‐presentation? Whilst research of IL‐6 trans‐presentation is required, much work has centred on the distinction between classical IL‐6R signalling and IL‐6 trans‐signalling. Here, the application of sgp130 has provided considerable evidence that IL‐6 trans‐signalling broadens the types of cells that become IL‐6 responsive (e.g. endothelial cells, fibroblasts, mesothelial cells, smooth muscle cells). The inflammatory regulation of sIL‐6R supporting a role for IL‐6 trans‐signalling in coordinating immune responses to disease. Classical IL‐6R signalling is, however, linked with the maintenance of immune or tissue homeostasis (e.g. regulation of the acute phase response, tissue regeneration, some metabolic processes). However, these distinctions are not unequivocal. For instance, classical IL‐6R signalling supports the expansion of IL‐17‐secreting CD4^+^ T‐helper cells, with sIL‐6R serving to retain circulating levels of IL‐6. Thus, there are shades of grey here, and the overall biological activity of IL‐6 is likely shaped differently in health and disease or different tissue compartments.

In the subsequent sections, we will now turn our attention to the intracellular mechanisms that influence the transmission of IL‐6 receptor signals from the cell membrane to the nucleus and affect the biological properties of IL‐6.

## The complexities linked to Jak‐STAT signalling

The signalling pathways associated with IL‐6 signalling are discussed at length in the literature [1, 4, 31, 32]. However, it is acknowledged that *context* is key to understanding IL‐6 signalling outcomes. This has been evidenced in models of bacterial peritonitis, a rare complication of peritoneal dialysis in patients with end‐stage renal failure [78]. Intraperitoneal administration of a patient derived isolate of *Staphylococcus epidermidis* in mice revealed that IL‐6 acting on surrounding stromal tissue initiates the recruitment of leukocytes into the inflamed peritoneum by inducing the expression of chemokines, cell adhesion molecules, acute‐phase‐reactants and processes related to tissue barrier permeability [79, 80, 81, 82, 83]. Here, IL‐6 is generated locally as a response to infection through the activation of stromal peritoneal cells (e.g. mesothelial cells) and resident mononuclear cells. However, this initial burst of IL‐6 requires an early influx of infiltrating neutrophils, which shed IL‐6R to promote IL‐6 trans‐signalling [84]. The release of sIL‐6R from the neutrophil cell surface promotes the IL‐6 regulation of genes linked to the control of neutrophil effector function, bacterial clearance and the resolution of acute inflammation [85, 86, 87, 88]. Subsequent, repeated bouts of inflammation distort this IL‐6 response and recurrent episodes of peritonitis promotes tissue remodelling and peritoneal fibrosis [83, 89, 90]. In this scenario, IL‐6 drives the proliferative expansion and retention of pro‐fibrotic interferon‐γ secreting CD4^+^ T cells within the local tissue [89]. This illustrative example showcases how IL‐6 responses adapt to changes in inflammation and emphasizes how IL‐6, working within a cytokine network, steers very different biological outcomes.

A key question is how IL‐6 family cytokines, sharing the same signal transducing subunit (namely gp130), can elicit unique STAT activation profiles and functional outputs. One explanation is likely to be found at the membrane interface of the cytokine‐receptor signalling complex. Here, our understanding rests on Cryo‐EM reconstruction of the extracellular region of the IL‐6‐IL‐6R‐gp130 hexamer [39]. Less clear, is how cytoplasmic portions of gp130 respond to receptor cross‐linking in partnership with different cytokine receptors and how these elicit different profiles of STAT transcription factor activation [36, 39, 45, 91]. We consider the example of IL‐6 and IL‐27. Both cytokines engage specific receptors (IL‐6R and IL‐27R) in partnership with gp130, but activate STAT1 and STAT3 to varying degrees, with different consequences [1, 92]. This is exemplified by observations in rheumatoid arthritis where the pro‐inflammatory role of IL‐6 contrasts with the more nuanced contribution of IL‐27, which inhibits the formation of ectopic lymphoid structures within the inflamed synovium [93]. Similar studies in T cells emphasize the opposing functions of IL‐6 and IL‐27 in determining the effector characteristics of CD4^+^ T cells [92, 93, 94, 95, 96]. At least in part, these differences are attributable to structural and sequence differences between the cytokine receptors. For example, mutation of tyrosine at position 613 to phenylalanine (Y613F) in the IL‐27 receptor cytoplasmic side chain enforces an IL‐6‐like STAT3 dominated phospho‐STAT response [97]. The Y613 residue serving as an additional docking site for STAT1. Time course analysis of IL‐27 signalling in Th1 polarized CD4^+^ T cells reveal that STAT1 activity confers an interferon gene expression signature *via* an IRF1 feed‐forward loop that is not seen in the transcriptional profile of IL‐6 [97]. Moreover, CD4^+^ T cells from *Stat1* ^−/−^ mice show that IL‐6 and IL‐27 induce very similar transcriptional programmes [98, 99]. Thus, changes in the threshold of STAT1 activation results in specific alterations in gene expression and includes impacts on the control of certain STAT3‐regulated genes [98, 99, 100, 101].

Alterations in the cytokine control of STAT transcription factor phosphorylation have important consequences at the gene level [45]. Studies employing chromatin‐immunoprecipitation in combination with next‐generation sequencing (ChIP‐seq) demonstrate how STAT transcription factors possess overlapping but not identical binding profiles that are not limited to canonical Gamma Activation Site (GAS)‐like motifs or Interferon‐regulated STAT‐responsive elements (ISRE) [45, 98, 100, 102]. These forms of genomic interaction or co‐occupancy contribute to both transcriptional cooperativity and the inhibition of gene regulation [45, 103, 104, 105]. It is also important to note that regulatory mechanisms acting on the immediate phosphorylation of STAT transcription factors also shape the transcriptional output of IL‐6.

The significance of these processes is evidenced as follows. Activated naïve CD4^+^ T cells isolated from *Stat1* ^−/−^ mice show increased STAT3 phosphorylation when stimulated with IL‐6 or IL‐27, while the reverse is true *Stat3* ^−/−^ mice [98]. As a consequence, the STAT3 binding profile is increased in response to STAT1 deficiency, and *vice versa* [98]. But what is the impact on gene regulation? This question is not easy to address, particularly as the relationship between STAT binding and gene regulation is not simply a matter of gene induction [100]. Transcriptomic analysis of *Stat3* deficient naïve CD4^+^ T cells shows that an absence of STAT3 results in a collapse in IL‐6 and IL‐27 gene regulation. In contrast, *Stat1* deficiency reduces the diversity of cytokine‐induced transcripts [98, 100]. These findings are further supported by studies in patients with STAT1 and STAT3 mutations and have led to the conclusion that while STAT3 is the principal driver of gene expression, STAT1 acts to fine‐tune the transcriptional output of cytokines such as IL‐6 and IL‐27 [98]. Therefore, it is not merely differences in phospho‐STAT activation profiles, but the precise regulatory function of each STAT transcription factor that determines cytokine‐specific gene expression.

## Fine tuning IL‐6 responses and the negative regulation of Jak‐STAT signals

**Fig. 2:**
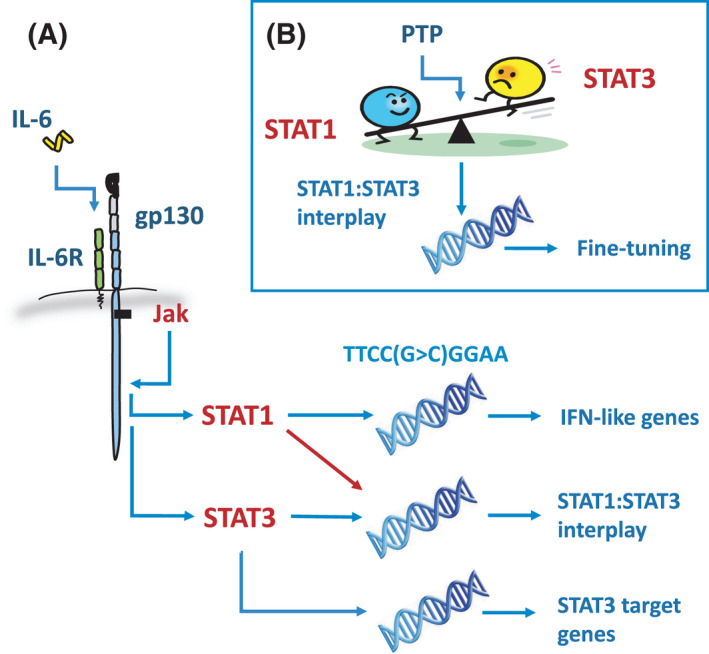
Relationship between STAT1 and STAT3 and IL‐6 responsiveness. (A) Jak‐STAT signalling in response to IL‐6 triggers activation of the latent transcription factors STAT1 and STAT3. The dimerization and translocation of these proteins to the nucleus targets genomic promoter and enhancer sites that include consensus DNA motifs for STAT transcription factor binding (e.g. IFN‐stimulated responsive elements; ISRE, IFNγ‐activated sequence; GAS). While STAT1 and STAT3 engage specific gene targets, with STAT1 controlling various interferon‐like responsive genes (e.g. *Irf1*, *Stat1* in CD4^+^ T cells) and STAT3 governing responses linked with metabolism, proliferation, survival, and functional identity. These often work at proximal and distal promoter sites. Extending studies originating from investigations in cancer, there is now increasing evidence supporting the role of STAT1 in shaping the transcriptional output of STAT3. (B) The balance of STAT1 and STAT3 activity is therefore instrumental in determining the transcriptional output of IL‐6 and regulatory mechanisms, including the action of protein phosphatases act to moderate or fine‐tune the cell response to IL‐6.

Competition for phospho‐tyrosine residues is not the only contributing factor in determining the output of Jak‐STAT signals. This is because STAT transcription factors are not thought to be present at saturating levels in the cytoplasm [45]. Instead, various regulatory mechanisms have evolved, which rate limit or fine‐tune the way cytokine cues are sensed and interpreted. These include the steric hindrance of Jak activity, the active identification of STAT transcription factors for de‐phosphorylation, and the post‐transcriptional targeting of cytokine mRNA [45]. For example, negative feedback inhibitors (e.g. the Suppressor of Cytokine Signalling protein family) that are induced by STAT transcription factors to dampen activation of the signalling cassette [45, 106]. Of these, SOCS3 is the best characterized. SOCS3 was first identified as an IL‐6 inducible pseudo‐substrate that binds Jak2 to terminate signalling [107]. SOCS3 binding centres on the recognition of a short Glycine‐Glutamine‐Methionine (GQM) motif on Jak2, which places SOCS3 in an orientation that directly blocks kinase docking to gp130 [31, 44, 108, 109]. This interaction prevents Jak binding to a specific phospho‐tyrosine residue with the gp130 sequence (identified at position 759 in man, and 757 in mice) [32, 46, 47]. SOCS3 also inhibits Jak1, Jak2, and Tyk2, but not Jak3, which lacks the GQM motif [47, 110]. However, it is the juxtaposition between Jak2 and gp130, that confers specificity of SOCS3 to IL‐6 family cytokines [111] (Fig. 2). The biological repercussions of SOCS3 inhibition have been evidenced in studies in *Socs3* ^−/−^ mice *in vivo* and patients lacking functional STAT1 and STAT3 transcription factors [47, 110]. SOCS3 deficiency shifts the IL‐6 regulated transcriptome towards an IFNγ‐like STAT1‐dominant response [112]. Moreover, quantitative real‐time PCR analyses have confirmed altered *SOCS3* transcript abundance in peripheral blood mononuclear cells from individuals with STAT1 gain‐of‐function and STAT3 loss‐of‐function mutations [113].

In addition to SOCS proteins, studies have shown that protein phosphatases also regulate Jak‐STAT cytokine receptor signalling. Examples include dual specificity protein phosphatases (DUSPs) and several PTPs [45, 114, 115]. For instance, in the multiple myeloma cell line U266, CD45 (termed PTP receptor type C; PTPRC) shapes the IL‐6 control of cellular adhesion and proliferation [116]. Significantly, these enzymes act as biological rheostats of cytokine signalling and function to fine‐tune the delivery or interpretation of inflammatory cues. Notably, DUSP2 inhibits STAT3 activation and restricts the generation of IL‐17‐secreting T‐helper cells (Th17 cells) in response to IL‐6 and TGFβ [114]. Conversely, PTPN2 and PTPN11 regulate the activities of STAT1 in fibroblasts [117, 118]. Whilst additional work is required to explain the functional specificity and selectivity of these enzymes for signalling intermediates within the Jak‐STAT cascade, studies suggest that changes in the cellular expression of these phosphatases alter the cytokine responsiveness of a cell. For example, transcriptional profiling of T cells shows that naïve CD4^+^ T‐cell activation increases the expression of several protein phosphatases, including PTPN2, PTPN22 and DUSP2. Thus, the transcriptional output of a cytokine maybe altered by changes in the expression of these enzymes. Studies have shown that naïve CD4^+^ T‐cell activation impairs the IL‐6 control of STAT1 in activated and memory CD4^+^ T‐cell subsets [83, 100, 119, 120]. This response is regulated by increases in the expression of PTPN2 (and to a lesser extent PTPN22) following T‐cell receptor activation [100]. Finally, the IL‐6 inducible RNase Regnase‐1 targets the poly‐adenylation tail of *IL6* mRNA as part of a post‐transcriptional negative feedback mechanism. Significantly, mice lacking the Regnase‐1 encoding gene (*Zc3h12a*) die prematurely of systemic auto‐inflammatory complications [121]. The physiological importance of these mechanisms is emphasised by a study showing that combined CRISPR targeting of *SOCS1*, *PTPN2*, and *Z3CH12A*, synergistically potentiates the anti‐tumour efficacy of CD8^+^ T cells by promoting effector memory T‐cell functions [122].

## MicroRNAs and their influence on IL‐6 receptor signals

**Fig. 3:**
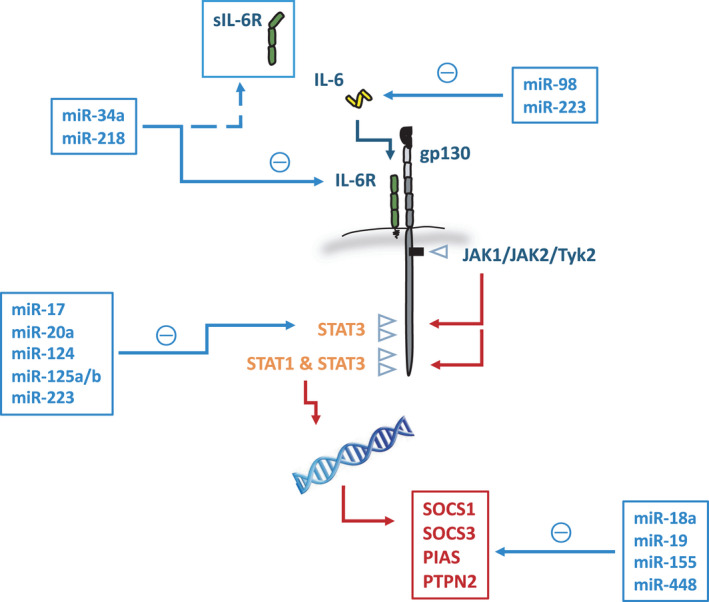
MicroRNA targeting of the IL‐6 receptor cassette. MicroRNAs with selectivity for components of the IL‐6 receptor and downstream signalling intermediates are shown. The dashed lines identify a potential influence on sIL‐6R bioavailability through the control of IL‐6R.

Various miR interfere with the IL‐6 signalling cassette. These regulatory elements fine‐tune the expression and bioavailability of specific components within the receptor system and Jak‐STAT pathway (Fig. 3). However, there is little evidence of direct targeting of IL‐6 by miR, due to the short 3′ untranslated region (3′ UTR) of the *IL6* transcript. The most compelling are miR‐98 and miR‐223 [123, 124, 125, 126]. In systemic lupus erythematous patients, a decrease in miR‐98 was negatively correlated with IL‐6 expression in peripheral blood mononuclear cells [123]. IL‐6 is also regulated by miR‐223. Studies in an animal model of excessive alcohol consumption show that IL‐6 expression is enhanced in *miR‐223* ^−/−^ mice and contributes to exacerbated ethanol‐induced hepatic injury through an in increase in neutrophil infiltration and reactive oxygen species generation [125]. A similar phenotype is also seen in skeletal muscle from *miR‐223* ^−/−^ mice where miR‐233 deficiency causes impaired muscle regeneration and increases in interstitial fibrosis due to the maintenance of a pro‐inflammatory macrophage population [124]. However, the miR‐223 binding site within the 3′ UTR of IL‐6 is not conserved in humans [127], which raises the question of relevance to disease.

Several miR target components of the IL‐6 receptor complex or signalling intermediates within the downstream Jak‐STAT pathway. For example, miR‐34 and miR‐218, which are identified as tumour suppressors [128, 129]. Critically, *miR‐34a* ^−/−^ mice show an increased incidence of colitis‐associated intestinal tumours that are typically larger in size and characterized by increases in IL‐6R expression and STAT3 activity [128]. These findings led to the development of a liposomal miR‐34a mimic (MRX34) for the treatment of cancer patients with solid tumours [130]. However, the Phase‐I clinical trials were terminated due to serious immune‐mediated adverse events resulting in four patient deaths [130]. Several other miR have been shown to target STAT3 (e.g. miR‐17, miR‐20a, miR‐124, miR‐125a, miR‐223) [131, 132, 133, 134]. With activities controlling the pro‐tumorigenic properties of the IL‐6 cytokine family, STAT3 drives various oncogenic processes affecting cell cycle progression and survival, angiogenesis, tumour invasion and metastasis [4]. In gliomas, STAT3 is a critical determinant of tumour associated immunosuppression and shows a negative correlation with miR‐124. Adoptive transfer of genetically engineered T cells expressing miR‐124 dampened STAT3 activity and enhanced anti‐tumour immunity [132].

The negative regulators of the Jak‐STAT cascade are also subject to miR‐mediated repression. miR‐155 and miR‐19 have been shown to target SOCS1 and SOCS3, respectively [135, 136]. In peritoneal macrophages, miR‐155 repression of SOCS1 enhanced interferon‐mediated anti‐viral immunity [135]. In gastric cancer, PIAS3 was shown to be repressed by miR‐18a, leading to increased expression of STAT3 transcriptional targets, such as BCL2L1 and MYC [137]. Lastly, miR‐448 repression of PTPN2 has been associated with Th17 cell differentiation in cerebrospinal fluids and blood mononuclear cells from patients with multiple sclerosis [138].

## Enhancer elements dictate the transcriptional output of IL‐6

STAT transcription factors rarely bind at gene promoter regions. More frequently, STAT binding occurs at intron‐localized motifs, sometimes intergenic regions, and often in close alignment with distal enhancer elements [45, 100, 139, 140]. Once thought to be relatively static determinants of cell lineages, enhancers are now appreciated to be dynamic regulators of gene expression that exhibit a high degree of plasticity through acetylation and deacetylation‐based mechanisms [102, 141, 142, 143]. Central to this plasticity, the histone acetyl transferase P300 (also termed CREB‐binding protein) is present at virtually all enhancers where it mediates transcription initiation at nearby promoters through the acetylation‐dependent recruitment of the transcription pre‐initiation complex and RNA polymerase II [142]. STAT transcription factor interactions with P300 are a well‐documented facet of the immune response where they promote H3K27 acetylation and enhancer activation [45, 100, 139, 140, 144, 145, 146, 147]. In a cytokine signalling context, regions of STAT‐P300 co‐occupancy may be viewed as sites of signal integration. For example, the generation of Th17 cells requires both IL‐6 and IL‐23 signalling, which combine to drive STAT3 and BLIMP‐1 binding to P300 enhancers at genes identifying a Th17 cell lineage [139, 144, 148]. A similar scenario is also seen in interferon‐γ‐secreting CD4^+^ T cells (Th1 cells) and IL‐4‐secreting CD4^+^ T cells (Th2 cells) where the localization of STAT4 and STAT6 to P300 enhancers promote the expression of the lineage defining transcription factors TBET and GATA3, respectively [139]. Thus, the interaction of STAT transcription factors with P300 sites shapes the effector properties and lineage fate of immune cells, including memory T‐cell subsets, macrophages and innate lymphoid cells [100, 139, 140, 144, 148, 149, 150].

Various biochemical studies have explored the interaction of STAT transcription factors with P300 [147, 151, 152, 153]. *In vitro*, STAT1 binding to P300 triggers a trans‐auto‐acetylation reaction, which catalyses the recruitment and activation of neighbouring P300 molecules. Here, the phosphorylation‐induced dimerization of the STAT1 transcription factor complex is important for bridging P300 molecules and drawing them into proximity with one another [147]. The crystallographic analysis of P300 identifies that these interactions are facilitated through acetylation reactions at functionally relevant domains within the autoinhibitory loop of P300 [147, 152]. These protein modifications contribute to the dynamic auto‐regulation of P300 activity when in proximity with activated STAT transcription factors [153]. This mechanism is transferable to other transcription factors (e.g. interferon responsive factors; IRF) [147].

Advances in next‐generation sequencing technologies (e.g. ChIP‐seq, ATAC‐seq and Hi‐C sequencing) are now affording exciting opportunities to understand how STAT transcription factors function in cooperation or competition at promoter sites (e.g. through cross‐regulation), and form meaningful partnerships with other transcription factors at enhancers. Here, *in silico* motif enrichment analyses and biochemical approaches support the idea that STAT transcription factors engage with highly dynamic enhancer interactomes in a context‐dependent manner [100, 140, 147, 154]. Thus, STAT proteins also perform scaffolding functions essential for the development of new enhancer architectures following stimulation. Nonetheless, further work is required to understand the spatial organization of STAT transcription factors within active chromatin regions.

ChIP‐seq and ATAC‐seq have evidenced the dynamic and complex nature of STAT transcription factor interactions with the genome (e.g. P300 sites as discussed) [45, 100, 139, 140, 144, 150]. These technologies have also identified the interaction of STAT transcription factors with latent enhancers, which are exposed following prior immune challenge with cytokines, pathogen‐associated molecular patterns, or damage‐associated molecular patterns [102]. Studies of macrophages stimulated *in vitro* have shown that the latent enhancer repertoire is ligand‐specific, delivering overlapping and distinct chromatin remodelling in response to different TLR ligands (CpG, MALP2, LPS) and cytokines (TNFα, IL‐1β, TGFβ, IFNγ) [102]. Latent enhancers are therefore sites of cytokine and TLR cross‐regulation with the potential to make nuanced contributions to inflammatory responses in the complex environment of tissue inflammation where numerous ligands act in concert. These forms of interaction may be relevant to cooperation seen between IL‐6/gp130 signalling and TLR2 and TLR4 in gastric tumours and models of septic shock [108, 155, 156, 157, 158]. Once exposed, these latent enhancers remain poised or active for some time [102, 149]. Latent enhancers are therefore described as a type of innate immune memory, which, unlike adaptive‐immune memory, preconditions cells for challenges they are yet to encounter [159]. Consequently, these enhancer sites might impact the stromal tissue response to infection, trauma or injury, and contribute to the development of inflammation‐induced tissue damage and chronic disease progression. Thus, genomic imprinting through enhancer re‐organization may contribute to a ‘genomic memory’ of a prior environmental stimulus. Here, it will be important to combine studies of genomic architecture with investigations of how cytokines like IL‐6 shape the proteome following inflammatory stimulation [97]. To understand this relationship, it is essential to identify the nature of these latent enhancers.

An emerging literature points to a subset of *Alu* elements functioning as either tissue‐specialized (poised/primed) or latent enhancers [160, 161, 162, 163]. *Alu* elements belong to a family of endogenous retroelements, which comprise up to 10% of the human genome and are often transcriptionally repressed through methylation [161, 164]. One study employed meta‐analyses of multiple publicly available genomics datasets to assess *Alu* enhancer function across tissues. This analysis identified involvements in processes integral to tissue specialization. These included neuronal involvement in the brain, immunological processes in the spleen, and metabolism in the Liver [160]. This functional analysis is supported by the highly tissue‐specific distribution of the ‘poised’ enhancer marker H3K4me1 at a subset of *Alu* elements with P300 occupancy. Thus, reflecting a proximity to genes controlling tissue identity [161]. These results are somewhat similar to functional characteristics of super‐enhancers, which were originally described as determinants of cell identity in terminally differentiated cells [141, 143]. Elsewhere, in a model of serum‐withdrawal, previously latent *Alu* enhancers were shown to promote cellular proliferation by regulating chromatin looping through interactions between RNA Polymerase III, general transcription factor III (TFIIIC), CCCTC‐binding factor (CTCF) and the cohesion complex [163]. Rather than acting in cis, *Alu* enhancers appear to govern the overall chromatin architecture influencing gene expression over long distances through the organization of three‐dimensional units of gene regulation termed topologically associated domains [161, 163]. Although this is yet to be investigated in an inflammation context, a role in coordinating higher‐order chromatin structure would fit neatly with the non‐linear relationship between intensity of cytokine‐receptor interaction, STAT transcription factor binding, and fold change in gene expression [100, 165]. Here, a potential link to the biology of STAT transcription factors is identified by the link between *Alu* elements and various interferonopathy‐like conditions [166, 167, 168]. We can, therefore, hypothesize that STAT transcription factor binding to *Alu* enhancer may coordinate *de novo* genomic architectures that facilitate adaptation to immune challenge. Further work is, however, required to characterize the definition of latent enhancers that are unveiled following exposure to immune or environmental challenge.

In summary, *in silico* motif enrichment analyses are supportive of the notion that STAT transcription factors form unique complexes in a highly context‐dependent manner [140]. Thus, while poised enhancers contain consensus sequences for STAT transcription factor binding, STAT‐linked latent enhancers are enriched for adenosine‐rich IRF binding sites [102]. For example, *Alu* enhancers are notable for their particularly high density of transcription factor binding motifs with at least one study proposing that evolutionary divergence has created *Alu* subsets that harbour distinct sets of concensus motifs [161]. Analysis of Encode deposited ChIP‐seq experiments has demonstrated binding of AP‐1 subunits (FOS, JUN, JUNB, JUND, FOSL1), SP‐1, GATA‐1, SMADs, and the chromosome looping co‐factor CTCF [160]. Other studies identify binding factors such as activity‐dependent neuroprotective protein, which bind almost exclusively to *Alu* enhancers [163].

## Relevance to IL‐6‐driven pathology

Genetic ablation studies in mice showcase roles for IL‐6 in both normal physiology and pathophysiology. These are often recapitulated in humans where biological drugs and small molecule inhibitors acting directly on the IL‐6 cytokine receptor or downstream signalling intermediates identify the importance of IL‐6 signalling through STAT1 and STAT3 [169, 170, 171, 172, 173].

The Jak‐STAT pathway has evolved to sense and interpret cytokine cues essential for cellular proliferation and functional identity [171]. These include activities controlling the maintenance of tissue homeostasis and host immunity against infection, autoimmunity and cancer [169]. As discussed, patients with genetic mutations affecting Jak‐STAT signalling display various immune deficiencies and biological drugs and small molecule inhibitors commonly prescribed in various disease settings often target STAT‐activating cytokines (e.g. or Jak activation) [1, 169, 171]. So, how does IL‐6 signalling through the Jak‐STAT pathway shape the course of the disease?

**Fig. 4:**
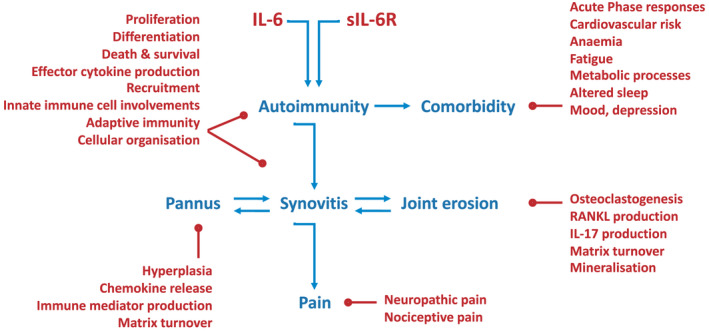
The complexities of IL‐6 biology in immune‐mediated disease. A summary of IL‐6 involvements in immune‐mediated inflammatory disease. Using rheumatoid arthritis as an example, the figure showcases the major contributions of IL‐6 to systemic immune outcomes, comorbidity, and reactions relevant to local pathology in the inflamed joint.

The transcriptional output delivered by the Jak‐STAT pathway is highly dynamic and incorporates partnerships with other transcription factors [45]. Working at transcriptionally relevant promoters or enhancers (as discussed earlier), these networks interpret cytokine cues responsible for disease progression. Here, gene regulation is often affected by a transcriptional interplay between individual STAT transcription factors (termed cross‐regulation) [45, 48, 101, 174, 175]. For example, STAT1‐activating cytokines show enhanced STAT3 responses in cells lacking STAT1 (and *vice versa*) [45]. Cross‐regulation is particularly evident in cancer cells where the deletion of STAT1 or STAT3 alters the expression of genes controlling tumour expansion and proliferative survival [48, 91]. While these genetic ablation studies illustrate how changes in STAT1 and STAT3 signals impact the fate or functional properties of a cell, the physiological processes steering cross‐regulation are unknown. However, these interactions may account for changes in leukocyte effector functions, the clinical presentation of pathology, and response to drug therapy. To contextualize the potential relevance of these mechanisms to disease, we draw on emerging literature from studies of rheumatoid arthritis and other autoimmune conditions (Fig. 4).

In mouse models of experimental autoimmune or inflammatory disease, IL‐6 deficiency often limits the histological signs of pathology. For example, hallmarks of synovial hyperplasia, synovial infiltration (or associated exudate), and joint erosion are all less pronounced in *Il6* ^−/−^ mice with arthritis [176, 177, 178, 179]. These outcomes reflect the impact of IL‐6 on cellular proliferation, the expression of chemokines, adhesion molecules and degradative enzymes, and bone turnover [180, 181, 182]. Despite the high concentration of IL‐6 in serum and synovial fluids of patients with arthritides, structural cells within the joint (e.g. chondrocytes, synoviocytes, fibroblasts, endothelial cells and activated infiltrating leukocytes) lack IL‐6R expression [100, 178, 183, 184]. In this regard, increases in synovial sIL‐6R correlate with extensive joint destruction and correspond with more advanced stages of rheumatoid arthritis [185, 186]. Thus, many of the biological outcomes associated with IL‐6 in synovitis are transmitted by IL‐6 trans‐signalling and require activation of the Jak‐STAT pathway and the latent transcription factor STAT3 [178, 184, 187, 188, 189].

The Jak‐STAT pathway senses and interprets cytokine cues targeted by drugs (e.g. tocilizumab, tofacitinib) commonly prescribed for the treatment of rheumatoid arthritis [1, 4, 45, 169, 170, 171]. As part of their mode‐of‐action, these inhibitors block cytokine signals elicited by STAT1 and STAT3 transcription factors [1, 169, 171]. In murine models, STAT1 activities often reduce arthritis severity [189, 190, 191], whereas STAT3 regulates leukocyte recruitment and activation, synovial hyperplasia and joint erosion [98, 178, 184, 192, 193, 194, 195, 196, 197, 198]. Genetic ablation studies show that STAT1 and STAT3 share a complex relationship, often opposing each other [45, 48, 91, 101]. For example, STAT1 shapes the transcriptional output of STAT3 in CD4^+^ T cells (e.g. altering effector functions) [45, 98, 99, 195, 197, 199, 200]. Studies in *gp130* ^Y757F:Y757F^ mice with antigen‐induced arthritis provide some evidence of STAT transcription factor cross‐regulation [184, 199, 201]. These animals possess a single tyrosine‐to‐phenylalanine substitution in the cytoplasmic domain of gp130 that causes a prolonged hyperactivation of STAT1 and STAT3 following cytokine activation [43, 44, 109, 201]. Here, *gp130* ^Y757F:Y757F^ mice with antigen‐induced arthritis displayed exacerbated joint pathology with synovitis revealing evidence of lymphoid aggregates [184, 199]. Moreover, analysis of CD4^+^ T cells from inguinal draining lymph nodes of *gp130* ^Y757F:Y757F^ mice with arthritis showed that genetic ablation of *Stat1* in these animals enhanced the expression of several STAT3 regulated cytokines involved in T cell‐driven synovitis [199]. Identical results were also observed in knock‐in mouse strains whereby the gp130‐dependent STAT3 and SHP2 signals were disrupted by replacing the mouse gp130 gene with human gp130 mutant cDNA (termed *gp130* ^Y759F:Y759F^) [42, 198, 202, 203]. The tyrosine residue at position 757 in mouse gp130 and position 759 in human gp130 is a critical regulatory determinant of gp130 signalling and is required for the recruitment of SH2‐domain containing cytoplasmic PTP SHP2 and the docking of SOCS3, which limits gp130‐mediated STAT transcription factor activation [46]. In this regard, the genetic manipulation of SOCS3 activity in experimental models of arthritis often show similarities with the pathological outcomes recorded in both *gp130* ^Y757F:Y757F^ and *gp130* ^Y759F:Y759F^ mice [204, 205, 206].

Extending the investigation of STAT1 and STAT3 cytokine signalling to wild‐type, *Il6* ^−/−^ and *Il27ra* ^−/−^ mice with antigen‐induced arthritis, studies show that these mice develop joint disease resembling the heterogeneous features of synovitis commonly seen in humans with rheumatoid arthritis [93, 100, 184, 199, 207, 208]. For example, *Il6* ^−/−^ mice develop a low‐inflammatory pathology (lacking an immune cell infiltrate) resembling fibroblast‐rich synovitis [176, 177, 178, 179, 184, 188, 207, 208]. This pathology contrasts with that of wild‐type mice, which present with a diffuse inflammatory infiltrate, and *Il27ra* ^−/−^ mice where the presence of organized ectopic lymphoid‐like structures (ELS) resembled lymphoid‐rich synovitis [93, 178, 184, 207, 208, 209, 210]. Reflecting on these results, IL‐6 and IL‐27 regulate STAT1 and STAT3 activities through receptor systems containing gp130 [4, 98, 211]. However, IL‐27 elicits a stronger STAT1 response and often blocks STAT3‐driven outcomes [4, 98, 99, 211]. Here, changes in the relative activation of STAT1 versus STAT3 may influence immune cell effector function, the response of stromal tissues to inflammation and activities that may alter clinical outcomes or the course of disease. So, how are these processes regulated?

As discussed, cytokine signalling *via* the Jak‐STAT pathway is controlled at multiple levels [45, 106, 115, 171]. These include negative feedback mechanisms (e.g. SOCS proteins) induced by STAT transcription factors to limit activation of the signalling cassette [45, 106]. Others act on phosphorylation events immediately following cytokine receptor engagement [45]. For example, PTPs involved in metabolic and immune regulation. Activities associated with these enzymes include control of kinases and transcription factors in the Jak‐STAT cascade [45, 100, 115, 116, 117, 212, 213, 214]. These include PTPN2 and PTPN22, which often possess genetic traits associated with increased susceptibility for autoimmunity [215]. Focussing on the biology of PTPN2 and PTPN22, recent studies suggest that these regulatory enzymes act as rheostats of Jak‐STAT cytokine signalling and re‐tune the way cells respond to cytokine cues transmitted by STAT1 and STAT3. Using CD4^+^ T cells from *Lck*‐Cre:*Ptpn2* ^fl/fl^ (T‐cell restricted deletion) and *Ptpn22* ^−/−^ mice, PTPN2 and PTPN22 have been reported to inhibit STAT1 tyrosine‐phosphorylation (pY‐STAT1) in IL‐6‐treated effector memory CD4^+^ T cells [100, 216]. When compared to IL‐6 responses in naïve CD4^+^ T cells, this form of STAT1 regulation altered the types of genes under STAT3 control in memory CD4^+^ T cells [100]. In murine models, PTPN2‐deficiency exacerbates disease through activities on follicular T‐helper cells, regulatory T cells and B cells [216, 217, 218, 219, 220, 221, 222]. For example, *Ptpn2^+/−^* mice develop synovitis showing enriched expression of synovial Th17 cells and ELS in the SKG model of autoimmune arthritis, consistent with the development of synovitis in *Il27ra* ^−/−^ mice with AIA [93, 219]. Moreover, analysis of IL‐6‐treated effector memory CD4^+^ T cells showed that PTPN2 inhibition of pY‐STAT1 enhances STAT3 gene regulation through promoters displaying P300 super‐enhancer architectures [100, 139]. These included cytokines (IL‐17A, IL‐21), transcription factors (Bcl6), checkpoint regulators (CD274) and chemokine receptors (CXCR4, CXCR5) linked with lymphoid‐rich synovitis [100]. Here, analysis of *PTPN2* in human synovial biopsies showed the highest expression in lymphoid‐rich synovitis [100]. Thus, PTPN2 contributes to the control of Jak‐STAT signalling in lymphocyte‐driven pathology affecting the composition, organization and activities of cells involved in ectopic lymphoneogenesis. In contrast, PTPN22 shows a more universal pattern of expression in fibroblast‐rich, myeloid‐rich, and lymphoid‐rich forms of synovitis [100]. This may reflect the role of PTPN22 in other hematopoietic populations (e.g. myeloid cells) and stromal tissues, which includes the inhibition of STAT1 signals in T cells, macrophages and fibroblasts [100, 139, 214, 218, 223, 224, 225, 226, 227, 228]. Collectively, these data suggest that the interpretation of cytokine cues sensed by a common receptor subunit (e.g. gp130) are fine‐tuned by PTP to direct alternate inflammatory outcomes.

A key question is whether IL‐6 utilizes different enhancer subtypes to affect its homeostatic and disease‐causing properties. Latent enhancers, as an adaptive response to environmental stimuli, might be expected to drive processes relevant to supporting tissue adaptation. Meanwhile, enhancers linked to IL‐6 homeostatic properties are likely to be to be constitutively active as homeostasis is ongoing under tonic signalling. Indeed, genes contributing to set biological processes often share common mechanisms of transcriptional control [229]. Functional genomics offer support for this hypothesis. For example, the initial description of latent enhancers specifically referenced wound healing as an enriched Gene Ontology term [102]. Moreover, there is some evidence to support the role of specific enhancer subtypes in human pathology. For example, regions of high‐density P300 binding, termed super‐enhancers (or stretch‐enhancers), are disproportionately associated with disease susceptibility loci compared with regular enhancers [230]. In this regard, the Jak inhibitor tofacitinib was found to alter the expression of genes commonly identified in genome‐wide associated studies and controlled by super‐enhancers [139].

The connection between STAT transcription factors and *Alu* enhancers is significant due to the historic association of retroelements with SNPs linked to most types of human pathology. For example, systemic lupus erythematosus and Sjörgren's syndrome, and human genetic disorders such as Aicardi‐Goutieres syndrome and familial chilblain lupus [166, 167]. These diseases are described as interferonopathies and are often associated with altered STAT transcription factor responses [167, 168]. Thus, pathophysiology may arise through changes in the interpretation of cytokine cues. This might arise through several mechanisms, including insertion‐linked mutagenesis, *Alu*‐RNA linked autoimmunity, or interference with cellular processes including ribosome function. Retroelements are not static, and have the capacity, once expressed, to interfere with cellular processes and in rare cases, reintegrate into the genome [164]. Here, structural variations caused by *Alu* insertions often contribute to hereditary disease, blood disorders and neurological conditions. In inflammation, *Alu* RNA expression is interferon inducible and has the capacity to activate intracellular Pattern Recognition Receptors including MDA5 and RIG‐1 to further propagate the immune response [231, 232]. It has been postulated that changes in *Alu*‐RNA expression may contribute to neuroinflammation and neurodegeneration in Alzheimer's disease, and the functional properties of peripheral blood cells isolated from patients with Systemic Lupus Erythematous (SLE) [167, 233]. Further work is however required to extend the links between genetic disease susceptibility, STAT transcription factor involvement in these diseases, and the epigenetic mechanisms driving disease onset.

## Conclusions and perspectives

Advances in cytokine biology increasingly identify novel mechanisms of cytokine involvement in physiology and pathophysiology. Studies in animal models and cell systems have now been aided by the widespread use of cytokine targeting therapies in patients with complex immune‐mediated conditions, infectious diseases and cancer. This is epitomized by studies of the Jak‐STAT pathway, which frequently combine methods common to the evaluation of cellular immunology, histology, functional genomics and the holistic analysis of the proteome and transcriptome. These approaches provide a high‐level understanding of how cells and tissues sense and interpret cytokine cues during health and disease. Various challenges, however, still remain. Focussing on the biology of IL‐6, there is a pressing need to understand how this major inflammatory cytokine contributes to normal physiology, competent anti‐microbial host defence, and the transition to pathophysiology and systemic chronic inflammation. Here, the estate agents (realtor) adage ‘Location, Location, Location’ takes on an important meaning and offers opportunities to understand how the systemic and local inflammatory context is shaped to promote disease susceptibility, heterogeneity in patient pathology, multimorbidity and response to drug therapy.
